# Comparison of the effectiveness of two educational interventions on sleep quality in older adults: a randomized clinical trial^*^

**DOI:** 10.1590/1980-220X-REEUSP-2022-0326en

**Published:** 2023-01-16

**Authors:** Khelyane Mesquita de Carvalho, Maria do Livramento Fortes Figueiredo, Nelson Miguel Galindo, Guilherme Guarino de Moura Sá, Cynthia Roberta Dias Torres Silva, Polyana Norberta Mendes

**Affiliations:** 1 Universidade Federal do Piauí, Departamento de Enfermagem, Teresina, PI, Brazil; 2 Instituto Federal de Pernambuco, Departamento de Enfermagem, Pesqueira, PE, Brazil.; 3 Instituto Federal de Pernambuco, Departamento de Enfermagem, Belo Jardim, PE, Brazil.; 4 Centro Universitário Santo Agostinho, Departamento de Enfermagem, Teresina, PI, Brazil.

**Keywords:** Age, Slee, Nursing Car, Educational Technology, Anciano, Sueño, Atención de Enfermería, Tecnología Educacional, Idoso, Sono, Cuidados de Enfermagem, Tecnologia Educacional

## Abstract

**Objective:**

to compare the effectiveness of an educational intervention mediated by a booklet with verbal nursing guidelines in improving sleep quality in older adults.

**Method:**

this is a randomized, single-blind clinical trial, carried out with 126 older adults, of which 62 were allocated in group 1, who received health education using an educational booklet, and 64 in group 2, who were exposed to health education with verbal nursing guidelines. Sleep quality was verified by the Pittsburgh Index, Epworth Sleepiness Scale and variable minutes that it takes to sleep. In order to compare the pre and post-tests, within the group, the Wilcoxon and chi-square tests were used. Status change was assessed using McNemar’s chi-square test. To compare groups, Mann-Whitney and chi-square were used. The significance level was 5%.

**Results:**

older adults in both groups showed improvement in sleep quality (p > 0.05) after the interventions. There was no statistically significant difference between the interventions.

**Conclusion:**

the educational intervention mediated by a booklet and verbal nursing guidelines were equally effective in improving older adults’ sleep quality. RBR-993xf7.

## RESUMO

**Objetivo:**

comparar a eficácia de intervenção educativa mediada por cartilha com orientações verbais de enfermagem na melhora da qualidade do sono de idosos.

**Método:**

ensaio clínico randomizado, simples-cego, realizado com 126 idosos, dos quais 62 foram alocados no grupo 1, que recebeu educação em saúde com uso de cartilha educativa, e 64 no grupo 2, que foram expostos à educação em saúde com orientações verbais de enfermagem. A qualidade do sono foi verificada pelo Índice de Pittsburgh, Escala de Sonolência de Epworth e variável minutos que leva para dormir. Para comparar os pré e pós-testes, intragrupo, utilizaram-se os Testes de Wilcoxon e Qui-Quadrado. A mudança de *status* foi avaliada pelo Teste Qui-Quadrado de McNemar. Para comparar grupos, utilizou-se Mann-Whitney e Qui-Quadrado. O nível de significância foi de 5%.

**Resultados:**

os idosos de ambos os grupos apresentaram melhora na qualidade do sono (p > 0,05) após as intervenções. Não houve diferença estatisticamente significativa entre as intervenções.

**Conclusão:**

a intervenção educativa mediada por cartilha e as orientações verbais de enfermagem foram igualmente eficazes na melhora da qualidade do sono de idosos. RBR-993xf7.

## RESUMEN

**Objetivo:**

comparar la efectividad de una intervención educativa mediada por un cuadernillo con orientaciones verbales de enfermería en la mejora de la calidad del sueño en ancianos.

**Método:**

ensayo clínico aleatorizado, simple ciego, realizado con 126 ancianos, de los cuales 62 fueron asignados al grupo 1, que recibieron educación en salud mediante cartilla educativa, y 64 al grupo 2, que fueron expuestos a la educación en salud con orientaciones verbales de enfermería. La calidad del sueño se evaluó mediante el Índice de Pittsburgh, la Escala de Somnolencia de Epworth y la cantidad variable de minutos que se tarda en dormir. Para comparar las pruebas previas y posteriores, dentro del grupo, se utilizaron las pruebas de Wilcoxon y Chi-Cuadrado. El cambio de estado se evaluó mediante la prueba Chi-Cuadrado de McNemar. Para la comparación de grupos se utilizaron las Pruebas de Mann-Whitney y Chi-Cuadrado. El nivel de significación fue del 5%.

**Resultados:**

los ancianos de ambos grupos mostraron mejoría en la calidad del sueño (p > 0,05) después de las intervenciones. No hubo diferencias estadísticamente significativas entre las intervenciones.

**Conclusión:**

la intervención educativa mediada por cuadernillo y orientaciones verbales de enfermería fueron igualmente efectivas en la mejora de la calidad del sueño de los ancianos. RBR-993xf7.

## INTRODUCTION

The impacts of poor sleep quality involve health issues, such as increased risk of falls, cognitive impairment, impairment of respiratory and cardiovascular function, with a consequent increase in hospitalizations and mortality^(1,2)^. Sleep quality impairment affects the general population, intensifying in the aging process.

Worldwide, studies indicate that 40% of people aged 60 years or older have sleep disorder (SD)^(3,4)^. the prevalence is equally high and varies between 35% and 40% of complaints related to sleep quantity and quality in the same age group^(5,6)^.

Having an adequate sleep pattern becomes challenging, not only because of determinants of the multidimensionality of aging, but because professionals do not prioritize the demands related to older adults’ sleep/rest. However, this problem requires priority in the health team’s conduct.

Regarding the possibilities for coping with it, studies point to the effectiveness of non-pharmacological interventions to minimize the interruption and/or induce sleep, in addition to educational strategies related to sleep hygiene^(7,8)^. Sleep hygiene, Cognitive Behavioral Therapy (CBT), acupuncture and physical activity are examples of non-drug interventions. The practice of sleep hygiene, which seeks to adjust the environment through behavioral changes, is the most used non-pharmacological intervention. Randomized studies point to positive and lasting impacts on the overall sleep quality index when using this intervention, such as reducing latency time and night awakenings^(9,10)^.

Health education in nursing consultations is pointed out as a non-pharmacological care strategy capable of promoting subjective improvement in older adults’ sleep quality^(11,12)^. In this context, the use of a booklet in health education practices encourages self-care, due to its easy access and execution, low cost and possibility of use in the absence of health professionals. Nursing uses technology based on the successful results of intervention studies on different topics, such as prevention of falls in the hospital environment, breastfeeding and home care^(13,14)^.

The quality of the intervention is important, as it must be done in such a way as to provoke an interest in older adults in changing their behavior. Therefore, comparing interventions becomes relevant, as it offers scientific support for decision-making in different contexts, contributing with realistic expectations in health interventions regarding healthy sleep habits.

Since health education is an inherent activity for nursing professionals, the indispensability of their active and comprehensive participation in the constant and effective educational process that prioritizes older adults’ sleep quality is highlighted in order to act early on the changes.

Given the above, the objective was to compare the effectiveness of an educational intervention mediated by a booklet with verbal nursing guidelines in improving older adults’ sleep quality.

## METHODS

### Study Design

This is a randomized controlled clinical trial, with two parallel groups, with a 1:1 allocation rate, conducted from January to July 2018. The Consolidated of Reporting Trials (CONSORT) for Randomized Trials of Nonpharmacologic Treatments was followed^(15)^.

### Population

The population consisted of 1,773 older adults, registered in the urban area of Primary Health Care in the municipality of Bom Jesus, Piauí, Brazil.

### Site

Data were collected during home visits to the area assigned to nine Basic Health Units (BHU) of Family Health Strategy (FHS) in the urban area of the municipality.

### Selection Criteria

The study included older adults, living in the urban area, assisted by FHS, with good cognitive status^(16)^ and Pittsburgh Sleep Quality Index (PSQI)^(17)^ greater than or equal to five points. The index corresponds to poor sleep quality or presence of sleep disturbance; therefore, the higher the index, the worse the sleep quality.

Older adults with low cognition, depressive symptoms according to the Geriatric Depression Scale^(18)^, self-reported hearing, visual or speech problems and those who were not available to participate were excluded. Likewise, older adults in continuous use of antidepressant, sedative and psychotropic medications, in addition to chronic use of alcohol, cocaine, crack and amphetamines, were excluded from the study, as they interfered with the outcome variable^(6)^.

### Sample Definition

The sample was calculated from the formula $\text{n}={\left(Z\alpha X\sqrt{p1xq1}+Z\beta X\sqrt{p2xq2}\right)}^{2}/{\left(p2-p1\right)}^{2}$ for comparison between groups^(19)^, which considered a confidence coefficient of 1.96, desired test power of 0.84, expected difference between groups of 25%, totaling 118 participants allocated into two groups. Due to the possibility of losses, 15% were added to the sample size for both groups so that 139 participants were recruited (69 for group 1 and 70 for group 2).

### Data Collection

Data were collected from January to March 2018. The primary outcomes were sleep quality and sleepiness, measured from PSQI^(20)^ and its parameters, which verifies sleep-related participant’s behavior, and the Epworth Daytime Sleepiness Scale^(17)^, which measures sleepiness. As secondary outcomes, demographic, economic and clinical characterization data of older adults were recorded.

The older adults assisted by FHS in the municipality of Bom Jesus were allocated into different groups. While group 1 (G1) received health education using an educational booklet, group 2 (G2) was exposed to health education with verbal nursing guidelines.

For randomization, the 36 microareas of the municipality’s FHS were stratified, which totaled the clusters to be randomized (18 for each group). For randomization, the alphabetical order of the microarea names was considered, which were previously obtained during a visit to the BHU. The random form, of the 18 microareas included in G1 and the 18 in G2, was carried out with the aid of R software. For this, it was established that the sequence of numbers (1 and 2) presented randomly by the software would determine allocation in G1 and G2.

Randomization by microarea allowed blinding with concealment of the group that older adults participated in and geographically distanced the participants, avoiding contaminating the sample.

Community Health Workers (CHW) and FHS nurses previously provided a list of older adults to be visited. The time for the home visit (HV) was scheduled by CHW in advance. Participants stayed in the quietest place in the house, chosen by the older adults themselves, with the researcher positioned in front of them, without the presence of family members, so as not to interfere with the answers. During the first HV, pre-intervention instruments were applied for data collection, and immediately after, the interventions were performed.

For the older adults in G1, a booklet called “*Durma bem e viva melhor*” was used. This constitutes educational gerontotechnology built from the Health Belief Model (HBM), validated in terms of content by expert judges and assessed as comprehensible by older adults, which has illustrated guidelines regarding sleep hygiene distributed over 25 pages^(21)^.

Intervention content organization took into account the four pillars of the HBM theory. The first allowed the reader to reflect on susceptibility to poor sleep quality. The second referred to perceived severity, aimed at recognizing the physical and physiological consequences of sleep deprivation. Perceived benefit, the third pillar of the HBM, was centered on guidance on sleep hygiene, related to environmental and behavioral factors on how to make the bedroom cozy to sleep better and perceive daily well-being. Finally, the fourth pillar, self-confidence to carry out the action, encouraged older adults to have a committed attitude^(21)^.

In the intervention operationalization with G1, the booklet was used, printed in the colored version, in open format: A4 – 29 x 20 cm^(21)^. This was leafed through while the illustrated guidelines about sleep hygiene were explained by the researcher, pointed out in the printed material and observed by older adults. At the end of the guidelines, the booklet was given to older adults.

For the older adults in G2, the guidelines on sleep hygiene were presented in the form of a dialogued exposition, without a booklet or any other image resource; however, they followed the same content based on the HBM and sequence presented to G1^(21)^.

The guidelines for both groups were passed on based on the beliefs of the theoretical model adopted in this study. Older adults were encouraged to realize their susceptibility to illness due to not sleeping well, the severity of the situation, when this basic human need is not met, health behaviors to prevent/treat the problem and its benefits and self-confidence to carry out sleep hygiene guidelines, despite the barriers. Visit duration in both groups was approximately 60 minutes for each older adult.

Eight weeks after the educational interventions, in the second HV, the data collection instruments were reapplied in both groups. This step followed the same format as the pre-intervention. The time interval was defined by guidance of insomnia consensus^(6)^.

To this end, we proceeded with the qualification of three nurses to carry out the post-intervention assessment and these professionals returned to the older adults’ house without knowing their allocations in G1 or 2, proceeding with the application of the instruments. Auxiliary researchers underwent theoretical and practical training in order to guarantee standardization for data collection.

### Data Analysis and Treatment

Non-compliance with the normal distribution of variables was checked using the Kolmogorov-Smirnov test, adopting a significance level of 5% and a 95% confidence interval for all tests. Softwares R version 3.5.1 and SPSS version 1.1.0 were used for data analysis. Qualitative variables were summarized by absolute (n) and relative (%) frequencies.

To compare older adults’ sleep quality older adults before and after the educational intervention, descriptive statistics were used, using medians and interquartile intervals, as measures of central tendency and dispersion, consecutively. For pre- and post-test comparison (intragroup), Wilcoxon was used for numerical variables and chi-square to compare the proportion for categorical variables. McNemar’s chi-square verified change in status of the groups’ categorical variables, separately. Comparison of the intergroup sleep quality index of numeric variables used Mann-Whitney, and categorical variables, the chi-square, for proportion.

### Ethical Aspects

This study complies with Resolution 466/12, using a consent instrument (Opinion 2,404,143, approved in 2017) registered on the Brazilian Clinical Trial Registry (ReBEC) platform, under protocol RBR-nº993xf7.

## RESULTS

**Figure 1.**
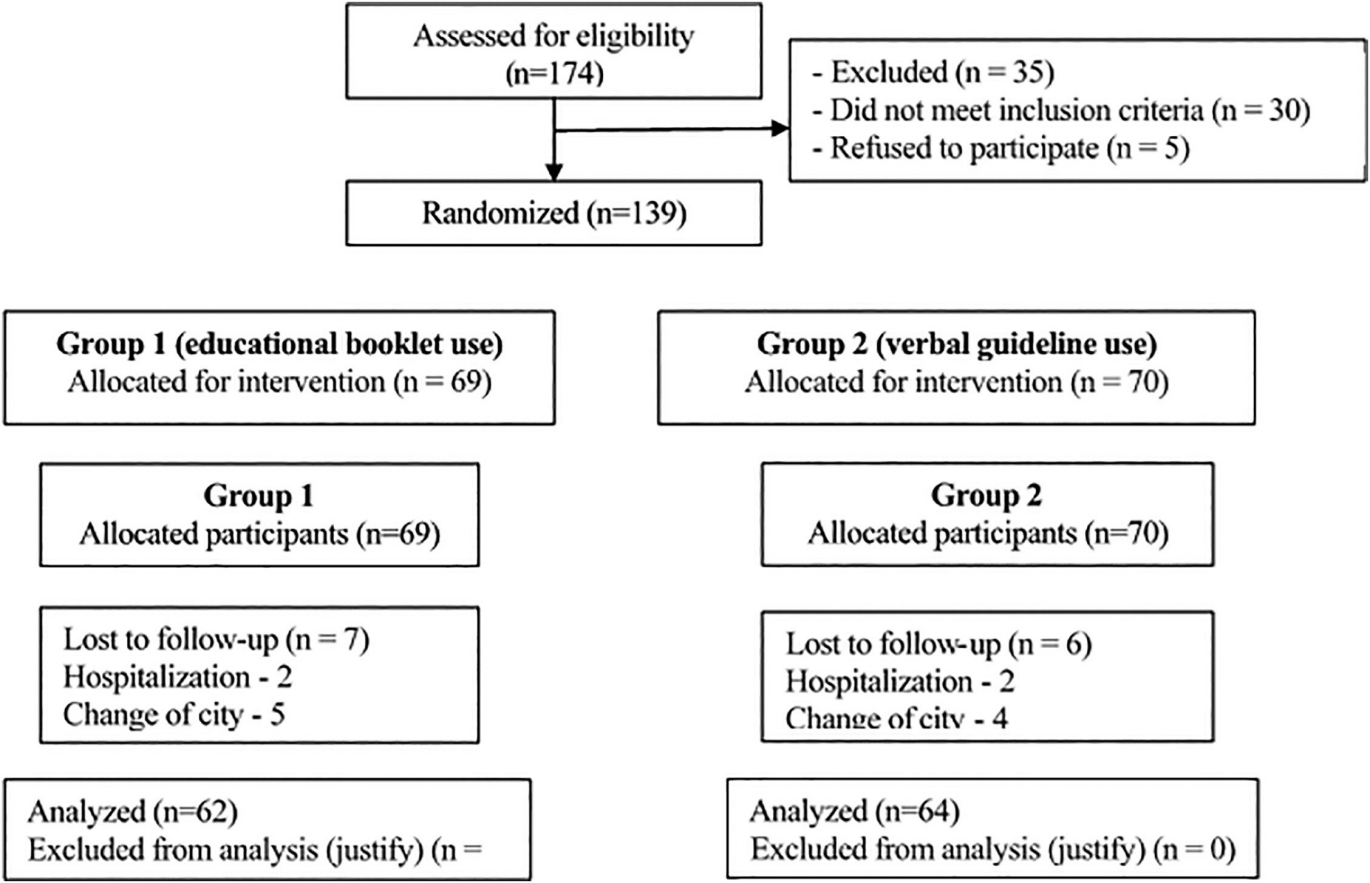
CONSORT flowchart of the randomized study. Bom Jesus, PI, Brazil, 2018.

A total of 174 older adults received HV, of which 139 were included in the study and allocated in groups according to randomization. In the eight-week follow-up, there were 13 losses, making a total sample of 126 participants who completed the study (G1 = 62; G2 = 64). Failure to meet the eligibility and discontinuity criteria are described below (Figure 1).

G1 and 2 were homogeneous for gender, age, skin color, religion and marital status. Most were women, aged between 60 and 70, Catholic and married. In terms of family arrangement, 45 older adults in G1 and 48 older adults in g2 reported living with their spouse, children, grandchildren and daughters-in-law.

Regarding the 62 older adults in G1, who were exposed to health education using an educational booklet, there was a change in efficiency of at least 85% of hours slept per night, present in 31 older adults in pre-test, increasing to 52 older adults after the intervention. Nocturnal awakenings to go to the bathroom per night reduced from 44 to 32 older adults who got up to go to the bathroom 3 or more times a night, per week, as shown in Table 1.

**Table 1.** Variables related to sleep quality parameters in group 1. Bom Jesus, PI, Brazil, 2018.

Variable	Pre-intervention		Post-intervention		p-value*
	N	%	N	%	
**Sleep efficiency/sleep hours per night (%)**					
> 85	31	50.00	52	83.87	**< 0.001**
75 to 84	12	19.35	8	12.90	0.503
65 to 74	6	9.67	2	3.22	0.289
< 65	13	20.96	0	0	^†^
**Waking up at night to go to the bathroom (per week)**					
None	6	9.67	5	8.06	1.000
Less than 1	6	9.67	11	17.74	0.227
1 or 2	6	9.67	14	22.58	0.077
3 or more	44	70.96	32	51.61	**0.004**
**Self-rated sleep**					
Very good	6	9.67	3	4.83	0.508
Good	24	38.70	50	80.64	**< 0.001**
Poor	29	46.77	8	12.90	**< 0.001**
Very poor	3	4.83	1	1.61	0.500
**Wake up in the middle of the night or early morning (per week)**					
None	0	0	5	8.06	0.063
Less than 1 time/week	3	4.83	8	12.90	0.180
1 or 2 times/week	7	11.29	11	17.74	0.388
3 or more times/week	52	83.87	38	61.29	**0.007**
**Concern as cause of sleep loss (per week)**					
None	15	24.19	30	48.38	**0.007**
Less than 1 time/week	20	32.25	7	11.29	0.011
1 or 2 times/week	7	11.29	13	20.96	0.263
3 or more times/week	20	32.25	12	19.35	1.907

*McNemar’s test; ^†^It was not possible to run McNemar’s test, as the contingency table used by the test had an entire column zeroed out.

In relation to self-rated sleep in this same group, the older adults who reported having good sleep were 24 in pre-test and increased to 50 in post-test. There was a reduction for those who self-rated sleep as poor from 29 before the intervention to eight after the intervention. Waking up in the middle of the night three or more times a week reduced from 52 older adults to 38. It is noteworthy that 30 older adults started not to worry weekly to the point of not sleeping after the intervention, according to Table 1.

Of the 64 older adults who were part of G2, exposed to verbal nursing guidelines, efficiency of at least 85% of sleeping hours was observed in 35.9% of older adults in the pre-intervention period, which increased to 82.8% after the intervention (p < 0.001). It is observed that there was a significant reduction in the number of older adults with sleep efficiency, being less than 65% of hours slept per night.

The variable “needed to go to the bathroom” was not statistically significant, but an improved behavior was observed in older adults who got up 3 or more times a night, with a reduction from 41 before the intervention to 34 after the intervention.

The perception of older adults’ sleep quality improved and older adults who self-rated sleep as good increased from 23 in the pre-intervention to 50 in the post-intervention. In the same perspective, there was also a significant reduction in the number of older adults who self-perceived sleep as poor and very poor, with a reduction from 29 (45.31%) to nine (14.06%) older adults and from seven (10.93%) to two (3.12%) older adults, respectively, according to Table 2.

**Table 2.** Variables related to sleep quality parameters in group 2. Bom Jesus, PI, Brazil, 2018.

Variable	Pre-intervention		Post-intervention		p-value*
	N	%	n	%	
**Sleep efficiency/sleep hours per night (%)**					
> 85	23	35.93	53	82.81	**< 0.001**
75 to 84	18	28.12	8	12.50	**0.004**
65 to 74	7	10.93	2	3.12	0.700
< 65	16	25.00	1	1.56	**< 0.001**
**Waking up at night to go to the bathroom (per week)**					
None	9	14.06	5	7.81	0.250
Less than 1	6	9.37	11	17.18	0.581
1 or 2	8	12.50	14	21.87	0.454
3 or more	41	64.06	34	53.12	0.481
**Self-rated sleep**					
Very good	1	1.56	3	4.68	0.625
Good	27	42.18	50	78.12	**< 0.001**
Poor	29	45.31	9	14.06	**0.002**
Very poor	7	10.93	2	3.12	0.070
**Trouble sleeping in up to 30 minutes (per week)**					
None	6	9.37	15	23.43	**0.049**
Less than 1 time/week	7	10.93	6	9.37	1.451
1 or 2 times/week	7	10.93	12	18.75	0.167
3 or more times/week	44	68.75	31	48.43	**0.006**
**Wake up in the middle of the night or early morning (per week)**					
None	2	3.12	5	7.81	0.063
Less than 1 time/week	2	3.12	8	12.50	0.687
1 or 2 times/week	6	9.37	11	17.18	0.077
3 or more times/week	54	84.37	40	62.50	**0.001**
**Concern as cause of sleep loss (per week)**					
None	7	10.93	30	46.87	**0.012**
Less than 1 time/week	19	29.68	7	10.93	1.558
1 or 2 times/week	13	20.31	13	20.31	1.000
3 or more times/week	25	39.06	14	21.87	0.064

*McNemar’s test, Brazil, 2018.

There was a significant improvement in relation to trouble sleeping for up to 30 minutes, since the number of older adults who reported having such difficulty three times a week or more fell from 44 in the pre-intervention period to 31 in the post-intervention period. Moreover, there was an increase in the number of older adults who reported having no trouble sleeping within 30 minutes, from six older adults before the intervention to 15 after it. It is important to highlight that the same occurred with older adults who woke up 3 or more times in the middle of the night or early in the morning, which reduced from 54 in the pre-intervention period to 40 in the post-intervention period.

As for the presence of concerns as a reason for not being able to sleep, it was observed that the intervention was effective, since, in pre-test, seven older adults reported not losing sleep due to worry and, in post-test, there was an increase to 30, as shown in Table 2.

Comparing the groups before and after the experiment, no statistically significant difference was observed. There is a significant intragroup observance for the reduction of latency time measured in minutes to sleep, in G1 and 2, when compared before and after the intervention. In the intergroup comparison, it is observed that sleep parameters (in minutes) were significant, according to Table 3.

**Table 3.** Comparison of the Pittsburgh Index, Epworth Sleepiness Scale and variable related to sleep quality in the two groups. Bom Jesus, PI, Brazil, 2018.

Variable Median	Group 1		Group 2		p-value*
	Median	(Min/Max)	Median	(Min/Max)	
**Pre-intervention**					
PSQI	19.5	(7.38)	19.5	(6.38)	0.467
Epwort	6	(0.21)	7	(0.21)	0.654
Minutes to fall asleep	60	(0.180)	85	(0.240)	**0.007**
**Post-intervention**					
PSQI	18.0	(8.35)	20	(8.35)	0.145
Epwort	8	(0.20)	8	(0.17)	0.730
Minutes to fall asleep	20	(0.180)	25	(0.240)	0.440
**Difference**					
PSQI_DIF	2	(–25.25)	2	(–14.15)	0.506
Epwort	–1	(–12.12)	–0.5	(–11.12)	0.428
Minutes to fall asleep	10	(–55.180)	40	(–180.24)	**0.028**

*Mann-Whitney test.

Regarding sleep quality parameters, as shown in Table 4, it is observed that there was no significant difference between the groups, which indicates that the interventions had similar effectiveness.

**Table 4.** Comparison of variables related to sleep quality parameters, in groups 1 and 2, before and after the intervention. Bom Jesus, PI, Brazil, 2018.

Variable	Pre-intervention			Post-intervention		
	Group 1 n (%)	Group 2 n (%)	p-value	Group 1 n (%)	Group 2 n (%)	p-value
**Hours of sleep per night**						
> 85%	31 (57.4%)	23 (42.6%)	0.111*	47.3%	58 (52.7%)	0.255*
75% a 84%	12 (40.0%)	18 (60.0%)	0.298*	8 (66.7%)	4 (33.3%)	0.203*
65% to 74%	6 (46.2%)	7 (53.8%)	0.816*	2 (66.7%)	1 (33.3%)	0.616^†^
< 65%	13 (44.8%)	16 (55.2%)	0.591*	0 (0.0%)	1 (100.0%)	1.000^†^
**Waking up at night to go to the bathroom (per week)**						
None of the time	6 (40.0%)	9 (60.0%)	0.447*	5 (45.5%)	6 (54.5%)	0.794*
Less than 1 time/week	6 (50.0%)	6 (50.0%)	0.954*	11 (55.0%)	9 (45.0%)	0.572*
1 or 2 times/week	6 (42.9%)	8 (57.1%)	0.614*	14 (53.8%)	12 (46.2%)	0.595*
3 or more times/week	44 (51.8%)	41 (48.2%)	0.408*	32 (46.4%)	37 (53.6%)	0.485*
**Self-rated sleep**						
Very good	6 (85.7%)	1 (14.3%)	0.060*	3 (50.0%)	3 (50.0%)	1.000^†^
Good	24 (47.1%)	27 (52.9%)	0.691*	50 (51.0%)	48 (49.0%)	0.446*
Poor	29 (50.9%)	28 (49.1%)	0.733*	8 (42.1%)	11 (57.9%)	0.502*
Very poor	3 (27.3%)	8 (72.7%)	0.128*	1 (33.3%)	2 (66.7%)	1.000^†^
**Trouble sleeping in up to 30 minutes (per week)**						
None	10 (62.5%)	6 (37.5%)	0.255*	15 (50.0%)	15 (50.0%)	0.921*
Less than 1 time/week	4 (36.4%)	7 (63.6%)	0.372*	6 (50.0%)	6 (50.0%)	0.954*
1 or 2 times/week	10 (58.8%)	7 (41.2%)	0.394*	12 (46.2%)	14 (53.8%)	0.727*
3 or more times/week	38 (46.3%)	44 (53.7%)	0.380*	29 (50.0%)	29 (50.0%)	0.869*
**Wake up in the middle of the night or early morning (per week)**						
None	0 (0.0%)	2 (100.0%)	0.496^†^	5 (41.7%)	7 (58.3%)	0.583*
Less than 1 time/week	3 (60.0%)	2 (40.0%)	0.677^†^	8 (66.7%)	4 (33.3%)	0.203*
1 or 2 times/week	7 (53.8%)	6 (46.2%)	0.724*	11 (44.0%)	14 (56.0%)	0.561*
3 or more times/week	52 (49.1%)	54 (50.9%)	0.938*	38 (49.4%)	39 (50.6%)	0.968*
**Concern as cause of sleep loss (per week)**						
None	15 (71.4%)	6 (28.6%)	0.056*	29 (61.7%)	18 (38.3%)	0.501*
Less than 1 time/week	20 (51.3%)	19 (48.7%)	0.755*	7 (30.4%)	16 (69.6%)	0.066*
1 or 2 times/week	7 (35.0%)	13 (65.0%)	0.166	13 (48.1%)	14 (51.9%)	0.901*
3 or more times/week	20 (43.5%)	26 (56.5%)	0.329*	13 (44.8%)	16 (55.2%)	0.591*

*Chi-square for proportion, ^†^Fisher’s exact test (when expected frequency < 5).

## DISCUSSION

The prevalence of worse sleep conditions was higher in female older adults. This finding may be related to the fact that women accumulate more tasks, such as raising children and grandchildren, which results in overload and dysfunctional thoughts, which culminate in changes in sleep architecture^(22,23)^.

In the present study, most older adults reported living with grandchildren, sons and daughters-in-law. A similar result found 39% of negative sleep perception in female older adults in this same family arrangement^(24)^. This fact may contribute to older adults’ poor sleep quality, due to changes in privacy and an environment that is not conducive to maintaining an adequate sleep pattern. The referred disadvantage emerges because restorative sleep requires attending to items such as a peaceful environment and with the least possible stimuli.

The results of this experimental study revealed an improvement in sleep quality in both groups (G1 and G2) after the interventions so that nursing guidelines and the use of the booklet were similarly effective in increasing sleep efficiency. This parameter is directly related to the number of hours slept and length of stay in bed, which also depend on the latency time and night awakenings, which consolidate its fragmentation and directly impact sleep architecture and organization.

The reduction in time to fall asleep converges with sleep hygiene guidelines anchored in the principles of the theoretical model of belief in health, in which older adults were encouraged to perceive the benefits of sleeping well and thus realize them. Successful results of nursing guidelines were observed in the states of Mato Grosso and São Paulo, also for older adults^(17,25)^. In this context, believing in action self-efficacy, i.e., believing in its ability to modulate factors that improve sleep, intensifies the correct practice, based on the reformulation of the idea that poor sleep is predictable in older adults^(26)^.

Sleep poor quality in people with dysfunctional beliefs requires an approach based on this theoretical model, which seeks to explain and predict the acceptance of recommendations on health care that prioritize the theme of sleep. Therefore, the HBM theory considers the stimuli, the uniqueness of the contexts, the knowledge of the population and anticipates that nurses should add strategies to their practices to provide adequate information, refute myths and misconceptions.

In the context of sleep fragmentation due to nocturnal awakenings, it is noteworthy that hypertensive patients increase cortisol and antidiuretic hormone secretion^(2)^. In this investigation, an average of 22 hypertensive older adults were identified in each group using diuretics, which, associated with the ingestion of many liquids at the end of the night, a particularly common practice among the older adults in the present study, contributes greatly to intermittent sleep voiding needs.

The HBM has been considered to explain and predict acceptance of health care recommendations. After the intervention, there was a reduction in the parameter of waking up in the middle of the night, as older adults took the position of ingesting less liquids close to bedtime as well as adjusting the time of diuretics. When verifying the effectiveness of this orientation and its possible impact on a sleep quality parameter, the belief in action effectiveness and the perception of its positive consequences stand out.

Self-rated sleep changed from a statistical point of view after both interventions. Such event, when contextualized in the light of the HBM, elucidates that the susceptibility to poor sleep was, in this study, reinforced by the belief that there is a condition/risk of falling ill and, consequently, under the belief of action effectiveness to reduce such risk. Similar to this situation, it shows the fact that the educational interventions carried out were dedicated to making older adults realize how serious it is not to sleep well and its consequences.

Self-rated sleep represents the way individuals see their state and understands their condition for illness, being considered a risk marker. In this study, some older adults self-perceived the risk of falling ill and compliance with sleep hygiene guidelines. This behavior was observed in studies carried out in Minas Gerais and Iran on the same subject^(1,24)^.

The preponderance of older adults who improved their perception of sleep self-rating after the experiment supports the idea that, when working on behavioral issues of the health belief model, it is possible to obtain good results from the perspective that older adults can sharpen their subjective perception of the personal risk of contracting a disease if they do not sleep and play a role in their self-care, made possible by the transfer of knowledge about the theme.

The results related to sleep efficiency converge to support significant changes: the reduction of minutes to sleep and, consequently, the reduction of trouble sleeping, which occurred significantly in both groups after different interventions. It is noteworthy that reducing the number of minutes to sleep is important, as insomnia can configure emotional destabilization in older adults, associated with anticipatory sleep anxiety^(6)^.

When it comes to older adults, poor sleep quality can result from erroneous habits and requires correction of maladaptive attitudes and beliefs that sleeping is luxury or laziness. These myths associated with the false belief that sleeping poorly in old age is predictable, reinforces to older adults that there is no effective treatment other than pharmacological.

Contrary to drug treatment, international studies have revealed that stimulus control, sleep restriction, sleep hygiene, cognitive therapy and relaxation techniques associated with health education are the first-line strategies, recommended for insomnia treatment in older adults due to lower risk of adverse effects and better quality of life^(27,28)^. This solidifies that building practices strengthen self-care.

Both interventions also modified parameters of sleep quality. Greater effects of the educational intervention mediated by the booklet may not have been obtained due to the influence of the low level of education that older adults had, as low education is a factor that contributes to worse health outcomes^(11,23)^.

A higher educational level is associated with health-beneficial behaviors and good results in understanding health information, which prevent predictive events of sleep problems. This fact may be related to a less sensitive improvement in sleep quality parameters of the group submitted to the intervention with an educational booklet, taking into account that older adults could feel discouraged from seeing the material due to the embarrassment of not knowing how to read, since the expressive majority attended little formal school and the material, despite being well illustrated, had text.

Verbal guidelines, without using printed material, proved to be sufficient to modify the reality found. It is pointed out that these, when they are part of the professional routine of empowered nurses, generate positive results by favoring significant learning by older adults, strengthening the user-professional relationship, promoting changes in life habits, exercising autonomy for healthy practices and reinforcing self-efficacy for changing behavior and overcoming perceived barriers, according to HBM.

It should be noted that health professionals should be encouraged to use the booklet, as well as to offer verbal guidance on sleep hygiene, as there was a change in important parameters of sleep quality, based on the two interventions used with the groups. Verbal guidelines present their importance and their need for use, especially for the public that will not be reached with a booklet, such as due to visual impairment.

One of the limitations of the study is the fact that it was carried out from older adults’ exposure to the intervention that occurred in a single moment (without repetitions). Finally, the assessment of the effect of the interventions took place in older adults in the community, Unified Health System (*Sistema Único de Saúde*) users, which may differ from the results obtained in interventions with institutionalized older adults or who are users of private health services.

## CONCLUSIONS

Educational intervention mediated by a booklet and verbal nursing guidelines without using the booklet were effective, considering the improvement shown in the post-test of the two groups so that, based on similar effectiveness, there was no difference between them, to improve older adults’ sleep quality during HV.
